# Notch-Hes-1 axis controls TLR7-mediated autophagic death of macrophage via induction of P62 in mice with lupus

**DOI:** 10.1038/cddis.2016.244

**Published:** 2016-08-18

**Authors:** Xiaojing Li, Fei Liu, Xuefang Zhang, Guoping Shi, Jing Ren, Jianjian Ji, Liang Ding, Hongye Fan, Huan Dou, Yayi Hou

**Affiliations:** 1 The State Key Laboratory of Pharmaceutical Biotechnology, Division of Immunology, Medical School, Nanjing University, Nanjing 210093, China; 2 State Key Laboratory of Natural Medicines, School of Life Science and Technology, China Pharmaceutical University, Nanjing 210009, China; 3 Jiangsu Key Laboratory of Molecular Medicine, Nanjing 210093, China

## Abstract

The increased death of macrophages has been considered as a pathogenic factor for systemic lupus erythematosus (SLE), and dysfunction of autophagy may contribute to improper cell death. However, the effect of autophagy on macrophage during the pathogenesis of SLE is still unclear. Here we found that the death rate and autophagy level of macrophages significantly increased in MRL/lpr lupus-prone mice. Activation of toll-like receptor 7 (TLR7) triggered macrophage death in an autophagy-dependent but caspase-independent way *in vitro*. Moreover, P62/SQSTM1 is thought to have an essential role in selective autophagy. We also demonstrated that P62/SQSTM1 was required for TLR7-induced autophagy, and knockdown of P62 suppressed R848-induced cell death and LC3II protein accumulation. As an important mediator for cell–cell communication, Notch signaling is responsible for cell-fate decisions. Our results showed that activation of TLR7 also upregulated the expression of Notch1, especially its downstream target gene Hairy and enhancer of split 1 (Hes-1) in macrophages. Of note, we found that Hes-1, as a transcriptional factor, controlled TLR7-induced autophagy by regulating P62 expression. Furthermore, to confirm the above results *in vivo*, TLR7 agonist imiquimod (IMQ)-induced lupus mouse model was prepared. Splenic macrophages from IMQ-treated mice exhibited increased autophagy and cell death as well as enhanced expressions of Notch1 and Hes-1. Our results indicate that Notch1-Hes-1 signaling controls TLR7-induced autophagic death of macrophage via regulation of P62 in mice with lupus.

Systemic lupus erythematosus (SLE) is a typical autoimmune disease with heterogeneous clinical presentations including skin rash, glomerulonephritis and leukopenia.^1^ Much of the pathophysiology and therapy of SLE has emphasized on autoimmune reactive B and T cells. Recently, focus has shifted to the role of macrophages in SLE pathogenesis.^2, 3, 4, 5^ Macrophages are innate immune system cells and have a pivotal role in removal of apoptotic and necrotic materials from tissues.^6^ Some studies reported that increased macrophage apoptosis occurred in SLE patients.^7, 8^ This leads to impaired clearance ability of SLE patients and accelerate autoimmunity.^9^

Autophagy is a catabolic process that enables cells to clean up unwanted or damaged cellular or non-self portions and degrade their constituents in a regulated manner.^10^ As is well known, the level of autophagy is elevated during intracellular and extracellular stress, such as starvation, hypoxia, bacterial and virus infection etc. Increased autophagy can help cells relieve stress and return to homeostasis through efficient removal of toxical factors produced during metabolic stress.^11, 12^ Although autophagy can serve to protect cells, uncontrolled upregulation of autophagy may lead to cell death.^13, 14^ Currently, malfunction of autophagy has been considered as a key component in the pathogenesis of SLE.^15, 16^ Genetic analysis studies discovered tight association between single-nucleotide polymorphism in autophagy-related gene 5 and SLE susceptibility.^17^ Moreover, increased level of autophagy was detected in B cells and T cells from murine and human lupus.^18, 19^ However, the role of autophagy in lupus macrophages is poorly understood.

During recent years, the pathogenic role of toll-like receptor 7 (TLR7) in SLE has been widely recognized. TLR7-deficient lupus-prone mice showed decreased lymphocyte activation and ameliorated disease symptoms.^20, 21^ In contrast, wild-type mice treated with TLR7 agonists developed severe systemic autoimmunity.^22^ Our previous studies also revealed a pathogenic role of TLR7 in SLE.^23, 24^ It was reported that TLR7 could detect single-stranded RNA and eliminate intracellular pathogens by inducing autophagy.^25^ In particular, some studies have indicated that P62/SQSTM1 has an essential role in selective autophagy induced by various stressors.^26^ P62/SQSTM1 is a member of sequestasome-like receptors, which acts as an adaptor by directly binding ubiquitinated bacteria (or probably viruses) and targeting them to autophagosomes.^27, 28^ However, the role of P62 in TLR7-mediated autophagy and the molecular mechanism underlies this process need to be further studied.

As an important mediator for cell–cell communication, Notch signaling is responsible for cell-fate decisions.^29^ In the immune system, Notch signaling was found to participate in regulating the activation and polarization of macrophages.^30, 31^ Accumulated data also suggest that aberrant gain or loss of Notch signaling components associated with various diseases, including osteoarthritis, experimental autoimmune encephalomyelitis and cancer and etc.^32, 33, 34^ Several groups recently have reported the diminished expression of Notch1 in T cells of SLE patients.^35, 36^ Of note, the expression of Hairy and enhancer of split 1 (Hes-1), one of the best-characterized downstream transcription factor of Notch signaling, also decreased in SLE T cells.^37^ And it has been reported that autophagy could regulate Notch signaling activity.^38^ However, it is still unclear whether Notch-Hes-1 axis could participate in onset or development of SLE through regulation of macrophage autophagy.

In this study, the roles of Notch signaling in TLR7-induced autophagic death of macrophage were examined. We found that both increased death and enhanced autophagy of macrophages occurred in MRL/lpr lupus-prone mice. TLR7-induced autophagic death of macrophages was dependent on P62/SQSTM1. Activation of TLR7 contributed to the upregulation of Notch1 signaling *in vitro* dramatically. Moreover, we also found that Hes-1 controlled TLR7-induced autophagy by regulating P62 gene expression at transcriptional level. Furthermore, we established another SLE murine model by treating C57BL/6 mice with TLR7 agonist imiquimod (IMQ) topically for 10 weeks. Expectedly, splenic macrophages from the mice exhibited increased autophagy and cell death. And the expressions of Notch1 and Hes-1 were also enhanced *in vivo*. These data indicate that Notch1-Hes-1 signaling can participate in the pathogenesis of SLE through regulation of TLR7-induced autophagic death of macrophage.

## Results

### Increased death and TLR7 expression of macrophages are exhibited in lupus mice

**Figure 1:**
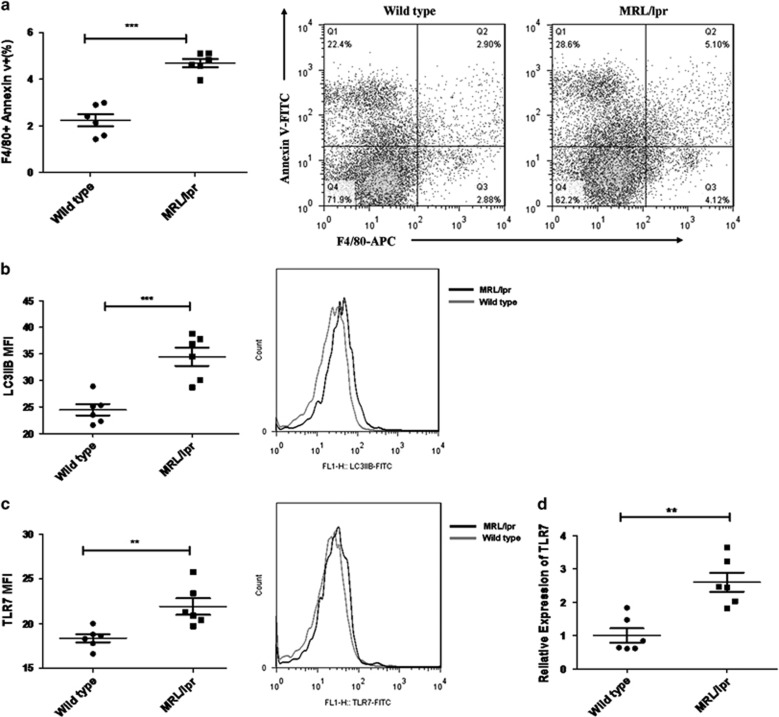
Increased death and TLR7 expression of macrophages are exhibited in lupus mice. Splenic cells and peritoneal macrophages were obtained from 22 week-old female MRL/lpr mice (*n*=6) and age matched female C57BL/6 mice (*n*=6). (**a**) The percentage of F4/80+Annexin V+ macrophages among total splenic cells was examined by flow cytometry (left). Representative FACS dot plots showed the proportion of F4/80+Annexin V+ macrophages among all of the splenic cells (right). (**b**) Flow cytometry analysis of LC3IIB level in F4/80+ splenic macrophages. One pair of representative histograms was shown on the right of the picture. (**c**) Quantification of TLR7 expression in F4/80+ splenic macrophages by FACS analysis (left). Representative histograms were shown on the right. (**d**) mRNA level of TLR7 in peritoneal macrophages from lupus mice and control mice was detected by real-time PCR. (**a**–**d**) The data are shown as means±S.E.M.s and represent two independent experiments. *P-values <0.05, **P<0.01 and ***P<0.001, determined by *t*-tests

Although increased macrophage apoptosis has been reported in SLE patients,^7, 8^ the apoptotic status of macrophages in lupus mice is still unclear. Thus, we examined the percentage of Annexin V+ macrophages in spleens of MRL/lpr lupus-prone mice at 22 weeks. Flow cytometry analysis results showed that the proportion of F4/80+ Annexin V+ macrophages increased significantly in lupus mice compared with healthy controls (Figure 1a). The level of LC3II, a classical marker for autophagy, enhanced significantly in splenic macrophages from lupus mice (Figure 1b). Meanwhile, the expression of TLR7 was also increased in splenic macrophages from lupus mice (Figure 1c). Moreover, real-time PCR results showed that the mRNA levels of TLR7 were significantly higher in peritoneal macrophages from lupus mice than those of control mice (Figure 1d). Taken together, these results indicate that macrophages in lupus mice displayed increased expression of TLR7, elevated death and enhanced autophagy.

### Activation of TLR7 contributes to macrophage death in an autophagy-dependent way

**Figure 2:**
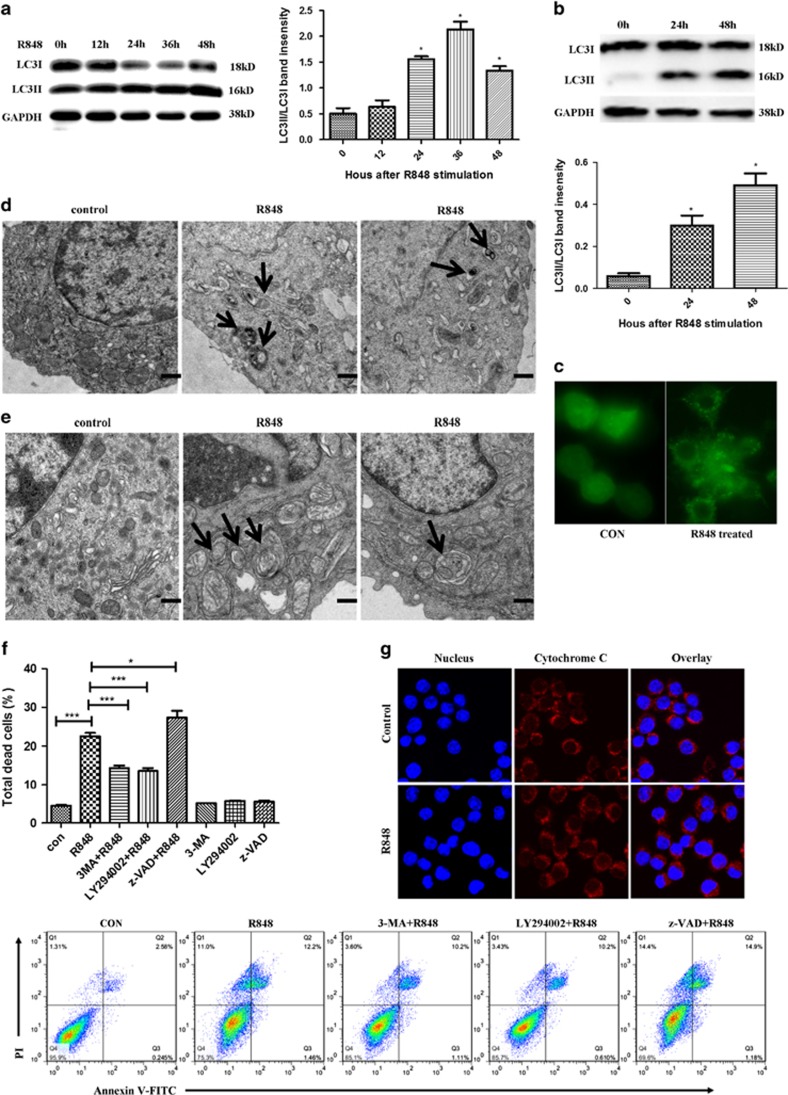
Autophagy is involved in R848-triggered macrophage death *in vitro*. RAW 264.7 cells and BMDMs were unstimulated or stimulated with 0.1 *μ*g/ml R848 or 0.01 *μ*g/ml R848, respectively. (**a**) Protein expression of LC3II in RAW 264.7 cells treated with R848 for indicated time was detected by western blot (left). Graphical representation of band intensities was shown on the right of the picture. (**b**) LC3II expression in BMDMs was examined by western blot after R848 stimulation for indicated time (top). Representative graph of band intensities was shown in the picture blow. (**c**) Fluorescent images of RAW 264.7 cells transfected with the pcDNA3.1 -GFP-LC3 plasmids and treated with R848 for 24 h subsequently (original magnification × 1000). The images showed GFP-LC3 dots formation in R848-stimulated cells. (**d**, **e**) Transmission electron micrographs of RAW 264.7 cells (**d**) and BMDMs (**e**) treated with R848 for 48 h (scale bars: 500 nm). The arrows indicated double-membrane structured autophagosomes containing degrading contents. (**f**) RAW 264.7 cells were pre-treated with the autophagy inhibitor LY294002 (10 *μ*M), 3-MA (0.25 Mm) or the pan-caspase inhibitor z-VAD (40 *μ*M), respectively for 2 h and then treated with R848 for 24 h. The percentage of total dead cells was detected by flow cytometry. Total dead RAW 264.7 cells included Annexin V+ PI+, Annexin V+PI− and Annexin V− PI+ cells. Representative FACS dot plots were shown in the picture below. (**g**) RAW 264.7 cells were treated with R848 for 24 h and immunostained for cytochrome c. Images were acquired with a confocal microscope (original magnification × 180). (**a**, **b**, **f**) The data are shown as means±S.E.M.s and represent three independent experiments. *P<0.05, ***P<0.001, determined by *t*-tests

To confirm whether activation of TLR7 induces macrophage autophagy, both bone marrow-derived macrophages (BMDM) and RAW 264.7 cells were treated with R848, a TLR7 ligand, *in vitro*. Accumulation of LC3II was detected in both cells, and the results showed that the protein levels of LC3II significantly increased in a time-dependent manner (Figures 2a and b). Moreover, GFP -LC3 puncta formation in RAW 264.7 cells was monitored by fluorescence microscopy. As shown in Figure 2c, GFP -LC3 dots were remarkably observed in R848-stimulated cells, whereas GFP -LC3 was diffusely disributed in control cells. Furthermore, the results of transmission electron microscopy showed that R848-treated macrophages had double-membrane structures with degrading contents (arrows), indicative of autophagosome formation (Figures 2d and e). Together, these results suggest that activation of TLR7 induces autophagy of macrophages.

As induction of autophagy has a role in both cell survival and death,^39^ macrophage death after R848 treatment was measured by Flow cytometry. Compared with control cells, R848-stimulated RAW 264.7 cells exhibited increased cell death (Figure 2f). To further explore the role of autophagy in the process of cell death, RAW 264.7 cells were treated with R848 in the presence or absence of autophagy inhibitors- 3-Methyladenine (3-MA) or LY294002. As expected, both 3-MA and LY294002 significantly reduced RAW 264.7 cell death. In contrast, z-VAD, the pan-caspase inhibitor, did not block R848-induced RAW 264.7 cell death, but enhanced cell death (Figure 2f). To investigate whether cytochrome c (Cyt c) release in R848-induced RAW 264.7 cell death, immunofluorescence staining of Cyt c was conducted. Consistent with control cells, the sub-cellular distribution of Cyt c in R848-stimulated cells displayed a punctuate pattern, indicating its location within the mitochondria (Figure 2g). These data suggest that activation of TLR7 contributes to macrophage death in an autophagy-dependent but caspase-independent way.

### P62/SQSTM1 is requisite for TLR7-induced autophagic death of macrophages

**Figure 3:**
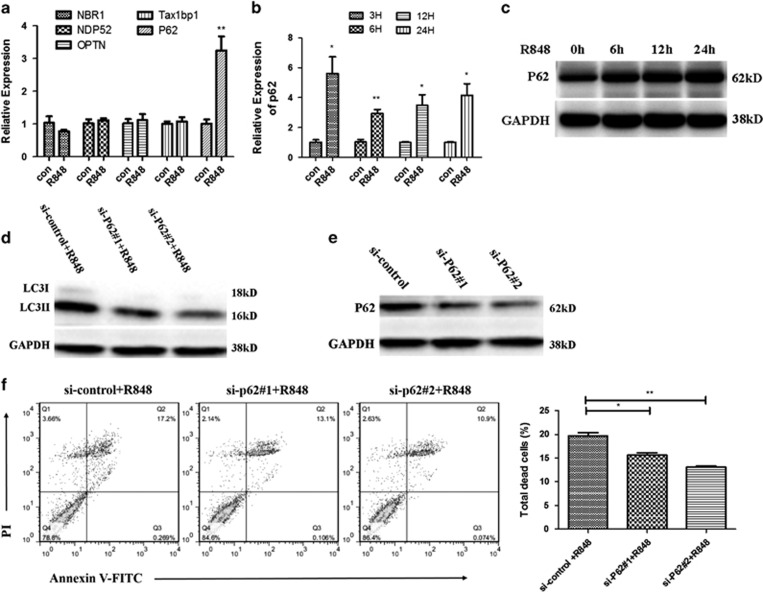
P62/SQSTM1 is requisite for TLR7-induced autophagic death of macrophages. (**a**) RAW 264.7 cells were unstimulated or stimulated with R848 (0.1 *μ*g/ml) for 6 h. Real-time PCR was carried out to examine mRNA levels of the five autophagic adaptors in RAW 264.7 cells. (**b**) P62 expression was examined by real-time PCR in RAW 264.7 cells after R848 (0.1 *μ*g/ml) stimulation. (**c**) Western analysis of P62 expression in RAW 264.7 cells treated with R848 (0.1 *μ*g/ml) for indicated time. (**d**, **f**) RAW 264.7 cells were transfected with si-control or si-P62 for 24 h and then stimulated with R848 for 24 h. si-P62#1 and si-P62#2 represent two independent siRNAs targeting P62. (**d**) Protein level of LC3II was detected by western analysis. (**f**) The percentage of total dead RAW 264.7 cells was detected by flow cytometry. Total dead RAW 264.7 cells included Annexin V+ PI+, Annexin V+PI−and Annexin V– PI+ cells. Representative FACS dot plots were shown in the left. (**e**) Degree of knockdown of P62 in samples used in (**d**). (**a**, **b**, **f**) The data are shown as means±S.E.M.s and represent three independent experiments. *P<0.05, **P<0.01, determined by *t-*tests

As single-stranded RNA (viral or non-viral origin) could trigger TLR7-mediated autophagy^25^ and autophagic adaptors have an important role in selective autophagy,^26^ we speculated that these adaptors may participate in TLR7-mediated autophagy. To examine which autophagic adaptor is involved in TLR7-mediated autophagy, RAW 264.7 cells were unstimulated or stimulated with R848 for 6 h. As shown in Figure 3a, R848-stimulated RAW 264.7 cells showed elevated expression of P62 but not that of other autophagic adaptor genes, suggesting a specific involvement of p62. Meanwhile, R848 treatment markedly enhanced mRNA and protein level of P62, but not in a time-dependent manner (Figures 3b and c). To further examine the role of P62 in TLR7-mediated autophagy, we knocked down P62 with two independent siRNAs. Suppression of p62 blocked R848-induced LC3II protein accumulation (Figures 3d and e). As our above results revealed that activation of TLR7 contributes to macrophage death in an autophagy-dependent way (Figure 2f), we tested whether P62 involves in TLR7-induced macrophage death. As shown in Figure 3f, knockdown of P62 resulted in a resistance to R848-induced RAW 264.7 cell death, confirming that TLR7-mediated auophagy indeed contributes to macrophage death. Collectively, these data suggest that TLR7-mediated autophagic death of macrophage is dependent on P62.

### Activation of TLR7 upregulates Notch1-Hes-1 signaling in macrophages

**Figure 4:**
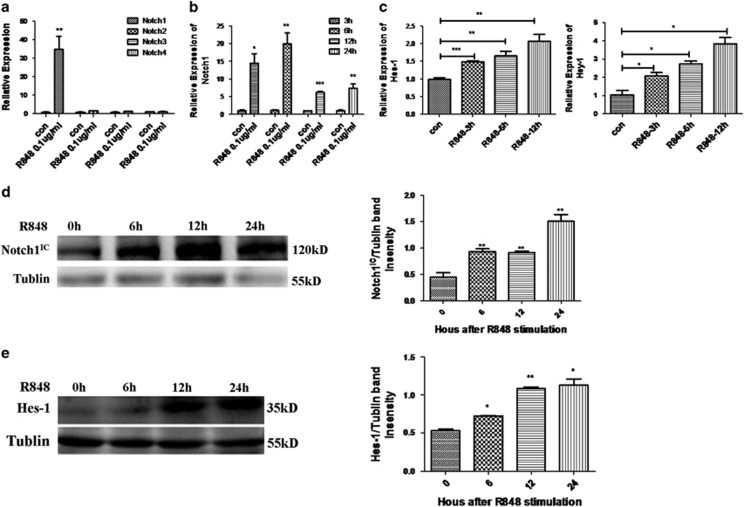
Activation of TLR7 upregulates Notch1-Hes-1 signaling in macrophages *in vitro*. RAW 264.7 cells were untreated or treated with 0.1 *μ*g/ml R848. (**a**) Real-time PCR analysis of Notch receptors expression at 6 h. (**b**, **c**) mRNA levels of Notch1 (**b**), Hes-1 and Hey-1 (**c**) in RAW 264.7 cells treated with R848 for indicated time. (**d**, **e**) Western analysis of protein expressions of Notch1^IC^ (**d**) and Hes-1 (**e**) in RAW 264.7 cells treated with R848 for indicated time. Representative graphs of band intensities were shown in the right of the blots. Notch1^IC^ expression was normalized to GAPDH and Hes-1 expression was normalized to Tublin. All data are shown as means±S.E.M.s and represent three independent experiments. *P<0.05, **P<0.01 and ***P<0.001, determined by *t*-tests

To date, four Notch receptors (Notch1-4) has been discovered.^40^ To investigate the effect of TLR7 stimulation on the expression pattern of Notch receptors in macrophages, RAW 264.7 cells were left unstimulated or stimulated with 0.1 *μ*g/ml R848 for 6 h. As shown in Figure 4a, R848-treated macrophages showed significant increase in the expression of Notch1 but not that of Notch 2, 3, 4. Moreover, real-time PCR and western blot results revealed that R848 stimulation markedly enhanced Notch1 expression, but not in a time-dependent manner (Figures 4b and d). Furthermore, downstream target gene of Notch1 signaling was examined. R848 stimulation increased the mRNA level of Hes-1 and Hey-1 in a time-dependent manner (Figure 4c). And protein level of Hes-1 was also upregulated by R848 stimulation (Figure 4e). Taken together, these data indicated that activation of TLR7 increases the expression of notch1, especially its downstream target gene Hes-1 in macrophages.

### Hes-1 regulates TLR7-mediated autophagic death of macrophages via induction of P62

**Figure 5:**
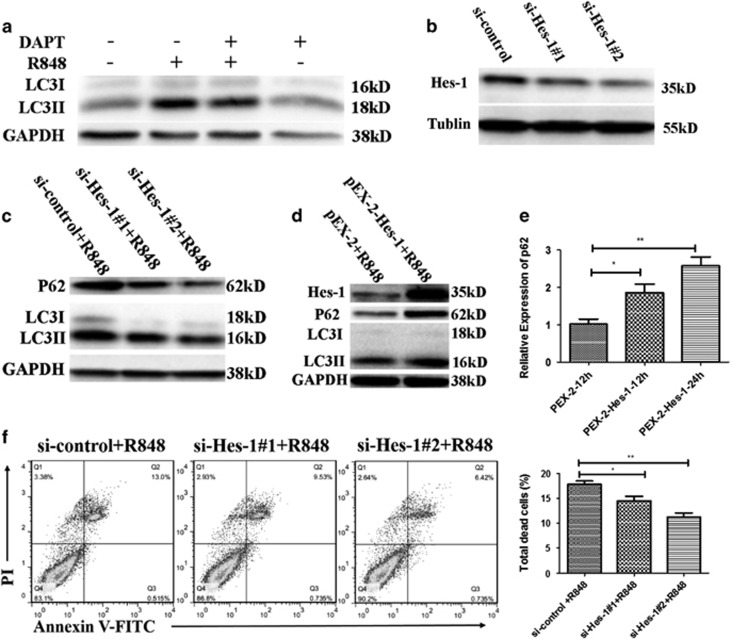
Hes-1 regulates TLR7-induced autophagic death of macrophages via P62. (**a**) RAW 264.7 cells were pre-treated with DAPT (10 *μ*M) or DMSO (0.02%) for 2 h and then stimulated by R848 (0.1 *μ*g/ml) for 24 h. Protein level of LC3II was assessed by western blot. (**b**) Degree of knockdown of Hes-1 in samples used in (**c**). (**c**, **f**) RAW 264.7 cells were transfected with si-control or si-Hes-1 for 24 h and then stimulated with R848 for 24 h. si-Hes-1#1 and si-Hes-1#2 represent two independent siRNAs targeting Hes-1. (**c**) Protein levels of Hes-1, P62 and LC3II were detected by western blot and normalized to GAPDH. (**f**) The percentage of total dead RAW 264.7 cells was detected by flow cytometry. Total dead RAW 264.7 cells included Annexin V+ PI+, Annexin V+PI− and Annexin V− PI+ cells. Representative FACS dot plots were shown (left). (**d**) PEX-2 plasmid transfected or PEX-2-Hes-1 plasmid transfected RAW 264.7 cells were stimulated with R848 (0.1 *μ*g/ml) for 24 h. Protein expression levels of Hes-1, P62 and LC3II were detected by western blot and normalized to GAPDH. (**e**) mRNA level of P62 in RAW 264.7 cells transfected with PEX-2-Hes-1 plasmid or control vector for indicated time. (**e**, **f**) The data are shown as means±S.E.M.s and represent three independent experiments. *P<0.05, **P<0.01, determined by *t-*tests

To evaluate the role of Notch1 signaling in TLR7-induced macrophage autophagy, RAW 264.7 cells were treated with Notch signaling inhibitor DAPT 2 h before R848 stimulation and the level of LC3II was examined. As is shown in Figure 5a, compared with control cells (R848 stimulation only), DAPT pre-treated-cells showed decreased level of LC3II, indicating an attenuation of autophagy. Hes-1 is the best-characterized target gene of Notch signaling. To determine whether Hes-1 is involved in TLR7-mediated autophagy, RAW 264.7 cells was transfected with si-control or two independent siRNAs against Hes-1 for 24 h and then stimulated with R848 for 24 h. Expectedly, Hes-1 knockdown not only decreased R848-induced LC3II accumulation (Figures 5b and c) but also suppressed R848-induced cell death (Figure 5f). To further validate the above results, Hes-1 was overexpressed in RAW 264.7 cells through pEX-2-Hes-1 plasmid transfection. After transfection with pEX-2-Hes-1 plasmids or control plasmids, the cells were treated with R848. As shown in Figure 5d, overexpression of Hes-1 markedly promoted R848-induced LC3II accumulation.

As our results showed that both P62 and Hes-1 were required for TLR7-induced autophagy, we thus speculate that Hes-1 may function as a transcriptional activator that directly regulates P62 gene expression. In line with expectations, overexpression of Hes-1 significantly increased P62 expression at mRNA level (Figure 5e) and enhanced R848-induced P62 expression at protein level (Figure 5d). Knockdown of Hes-1 reduced R848-induced P62 expression at protein level (Figure 5c). These results suggest that Hes-1 controls TLR7-mediated autophagic death of macrophages by regulating P62 expression at transcriptional level.

### Autophagic death of macrophage is re-verified in TLR7 agonist-induced lupus mouse model

The TLR7 agonist IMQ-induced lupus mouse model was prepared as previously described.^22^ The results revealed that IMQ-treated mice exhibited lupus-like symptoms with marked splenomegaly (Supplementary Figure 1A, 1B) and severe proteinuria (Supplementary Figure 2C). The activation marker CD69 was markedly upregulated in T cells and B cells of IMQ-treated mice (Supplementary Figure 1C, 1D). Hematoxylin & eosin (HE) staining of renal tissues revealed increased cellularity and severe glomerulonephritis in IMQ-treated mice (Supplementary Figure 2A, 2B).

**Figure 6:**
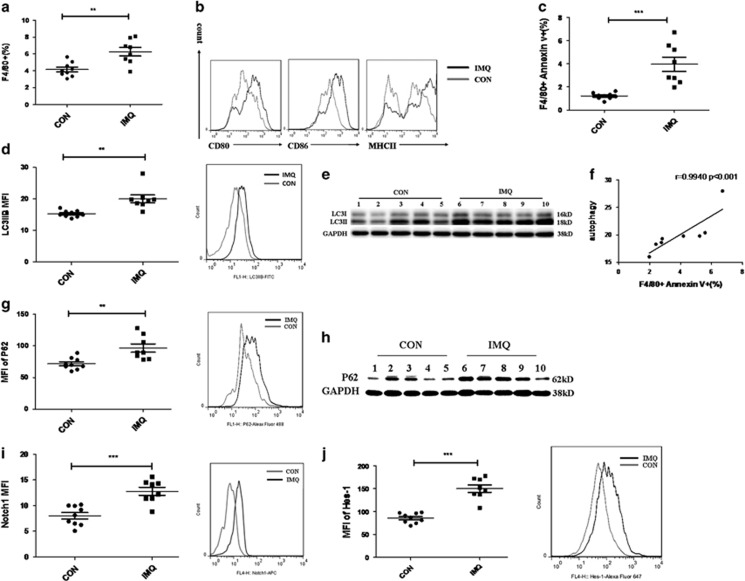
Autophagic death of macrophage is re-verified in TLR7 agonist-induced lupus mouse model. TLR7 agonist-induced lupus mouse model was established by treating wild-type C57BL/6 mice with imiquimod for 10 weeks. (**a**) The percentage of F4/80+ macrophages among total splenic cells in IMQ-treated mice (*n*=8) and control mice (*n*=9) was determined by flow cytometry analysis. (**b**) Expression levels of macrophage activation markers (CD80, CD86 and MHCII) in splenic macrophages from IMQ-treated mice (*n*=8) and control mice (*n*=9). (**c**) The proportion of F4/80+ Annexin V+ macrophages among total splenic cells in IMQ-treated mice (*n*=8) and control mice (*n*=9). (**d**, **g**, **i**, **j**) Flow cytometry analysis of the levels of LC3IIB (**d**), P62 (**g**), Notch1 (**i**) and Hes-1 (**j**) in splenic macrophages from IMQ-treated mice (*n*=8) and control mice (*n*=9). Representative histograms showed the MFI of detected proteins were all on the right of stastical charts. (**e**, **h**) BMDM cells were generated from the bone marrow of IMQ-treated mice (*n*=5) and control mice (*n*=5). The protein levels of LC3II (**e**) and P62 (**h**) were assessed by western blot and normalized to GAPDH. (**f**) The correlation analysis between percentage of F4/80+ Annexin V+ splenic macrophages and MFI of LC3II in splenic macrophages from IMQ-treated mice. And the correlation results were determined by linear regression analysis. (**a**, **c**, **d**, **g**, **i**, **j**) The data are shown as means±S.E.M.s and represent two independent experiments. **P<0.01, ***P<0.001, determined by *t*-tests

The presence of F4/80+ macrophages was assessed in spleens of control mice and IMQ-treated mice. Expectedly, the percentage of F4/80+ cells (Figure 6a) and the expression of their activation markers, including MHCII, CD80 and CD86 (Figure 6b) were increased significantly in IMQ-treated mice compared with control mice. Consistent with the results *in vitro*, the proportion of F4/80+Annexin V+ macrophages also increased markedly in IMQ-treated mice (Figure 6c). Meanwhile, the LC3II levels in both splenic macrophages and BMDMs from IMQ-treated mice also elevated significantly (Figures 6d and e). Moreover, there was a significant correlation between the LC3II level and the death rate of macrophages from spleens of IMQ-treated mice (Figure 6f). Of note, the level of P62 in macrophages from IMQ-treated mice also elevated significantly (Figures 6g and h). Furthermore, as shown in Figures 6i and j, IMQ treatment upregulated Notch1-Hes-1 signaling in splenic macrophages *in vivo*. Collectively, these data confirm that Notch1-Hes-1 signaling indeed controls TLR7-induced autophagic death of macrophage via regulation of P62.

## Discussion

It was reported that patients with SLE had increased apoptotic macrophage,^7, 8^ which may lead to defect in clearance of apoptotic cells and accelerate autoimmune disorders.^9^ However, the mechanism of enhanced macrophage death in SLE patients and lupus-prone mice need to be fully defined. In our present study, we observed that both the death rate and the autophagy level of macrophages from MRL/lpr lupus-prone mice were significantly enhanced. To further explore this phenomenon under SLE background, we treated RAW 264.7 cells and BMDMs with R848 *in vitro*. We demonstrated that activation of TLR7 signaling promoted macrophage autophagy by western blot analysis of LC3II expression, transmission electron microscopy observation of autophagosome and fluorescence microscopy detection of GFP-LC3- dots. Moreover, autophagy inhibitor could block macrophage death caused by R848 treatment, whereas the pan-caspase inhibitor could not. These results showed that TLR7 signaling could trigger autophagic death of macrophages. Next, RNA interference experiments demonstrated that TLR7-induced autophagy depended on P62 expression. Furthermore, our study showed that Hes-1, the best-characterized target gene of Notch signaling, was upregulated by R848 stimulation, and directly increased P62 expression at transcriptional level. At last, we generated a TLR7 agonist-induced lupus mouse model, and confirmed that Notch-Hes-1 axis controlled TLR7-mediated autophagic death of macrophages via induction of P62.

Although autophagy is mainly considered to have a cytoprotective role, uncontrolled upregulation of autophagy can also lead to cell death.^13, 14^ Dysfunction of autophagy in SLE has been reported.^18, 19^ We speculated that autophagy may be linked to increased macrophage death in SLE. We demonstrated that TLR7-induced autophagy could be a critical factor that contributed to increased macrophage death. TLR7 can recognize ssRNA from exogenous viruses, and exogenous viruses can induce autophagy.^41^ Thus, exogenous viruses may induce excessive autophagy and contribute to increased macrophage death in SLE. Further studies for specific virus infection are still needed in the future.

It was reported that P62 can function as an autophagic adaptor by directly binding ubiquitinated bacteria (or probably viruses) and targeting them to autophagosomes.^27, 28^ Moreover, P62 was known to have an important role in selective autophagy induced by bacteria and virus infection.^26^ Several studies have reported that P62 has an essential role in TLR4-mediated autophagy.^42, 43^ In addition, p62 also has a pro-survival role in huntingtin-induced Hella cell death.^44^ In contrast, P62 expression promoted resveratrol-induced myelogenous Leukemia cells death.^45^ However, whether P62 could regulate TLR7-mediated autophagic death of macrophages remains unknown. We demonstrated that P62 knockdown could attenuate autophagic death of macrophages caused by R848 stimulation. We also found that the level of P62 increased significantly in macrophages from IMQ-treated mice. Thus, it could be reasonable to infer that P62 may control macrophage death in SLE.

Notch signaling participates in many aspects of developmental processes and tissue renewal.^46^ In the immune system, Notch signaling has an essential role in T- and B-cell lineage commitment, Th1/Th2 differentiation^47^ and functional modulation of Tregs.^48^ Notch signaling promoted programmed cell death during erythropoiesis.^49^ More importantly, Notch signaling regulated cell death in a context-dependent manner and implicated in macrophage activation and polarization.^30, 31^ Besides, Notch signaling inhibitor DAPT could enhance curcumin-induced esophageal cancer cell death,^50^ whereas overexpression of NICD1 (Notch1 intracellular domain) in HEK and SH-SY5Y cells could promote cell death under ischemic conditions.^51^ However, it is still unclear that whether Notch signaling is involved in regulating macrophage death caused by excessive TLR7 activation. We found that Hes-1 (target gene of Notch signaling) could control TLR7-mediated autophagic death of macrophage by directly regulating P62 gene expression. As aberrant expression of Notch1 signaling components in SLE patients has been reported, we examined the levels of Notch1 and Hes-1 in IMQ-treated mice. The levels of Notch1 and Hes-1 increased significantly in IMQ-treated mice compared with those of control mice. Thus, these data suggest that Notch signaling may participate in SLE pathogenesis by controlling P62 transcription.

In summary, our investigation demonstrated that excessive and persistent activation of TLR7 could result in increased autophagic death of macrophages in SLE. Elevated Notch1 signaling promoted TLR7-mediated autophagic death of macrophages in the pathogenesis of SLE. Our study provides a new explanation for increased macrophage death during the pathogenesis of SLE. These data suggest that activation of TLR7 by environmental factors may bring about the abnormality of macrophages in SLE.

## Materials and methods

### Mice

Female MRL/lpr mice and age-matched female C57BL/6 mice were purchased from model animal research center of Nanjing University and killed at 22 weeks old. TLR7 agonist-treated mice with lupus-like syndrome were prepared as previously described.^23^ In brief, 7-9-week-old female C57BL/6 mice were treated topically with 5% IMQ cream (3M Health Care, MN, USA) to the ears three times weekly. The mice were killed after 10 weeks IMQ treatment. All the mice were housed in specific pathogen-free conditions at the Nanjing University Animal Care Commission.

### Chemicals and ligands

LY294002 and z-VAD-fmk solution were purchased from Beyotime, Shanghai, China. R848 (Enzo Life Science, Farmingdale, NY, USA) and 3-MA (Sigma, Shanghai, China) was prepared in ddH_2_O and DAPT (Merk Millipore, Bedford, MA, USA) was prepared in DMSO.

### Cell culture

RAW 264.7 cells were cultured in Dulbecco's modified Eagle medium (Gibco, Grand Island, NY, USA) supplemented with 10% FBS (Gibco) at 37 °C in a humidified atmosphere with 5% CO2. For generation of mouse BMDM, bone marrow (BM) cells were obtained from 8-week-old female C57BL/6 mice. BM cells were cultivated in RPM1640 medium (Gibco) supplemented with 50 ng/ml M-CSF (PEPROTECH, Rocky Hill, USA), antibiotics and 10% FBS. The medium was replaced on day 3 and the cells were collected and used on day 5.

### Plasmid construction and cell transfection

Mouse Hes-1 was chemically synthesized and cloned into pEX-2 vector, resulting in Hes-1-containning pEX-2 plasmid (pEX-2-Hes-1 plasmid, Genechem, Shanghai, China). RAW 264.7 cells were cultured in DMEM medium supplemented with 10% FBS in 24-well plates overnight and transfected with the pEX-2-Hes-1 plasmid (0.5 *μ*g/well) using the X-treme GENE HP DNA transfection reagent (Roche, Basel, Switzerland). Empty plasmid was transfected as matched control. The small-interfering RNA used for silencing mouse P62 and Hes-1 were obtained from Ribobio (Guangzhou, China). The RNAi sequences are as follows: For mouse P62#1, FP: 5-GCUGAAACAUGGACACUUU-3, RP:5-AAAGUGUCCAUGUUUCAGC-3; For mouse P62#2, FP:5-GCACAGAAGACAAGAGUAA-3, RP:5-UUACUCUUGUCUUCUGUGC-3; For mouse Hes-1#1, FP: 5-GGCAUUCCAAGCUAGAGAA-3, RP: 5-UUCUCUAGCUUGGAAUGCC-3; For mouse Hes-1#2, FP: 5-AGAUCAACGCCAUGACCUA-3, RP: 5-UAGGUCAUGGCGUUGAUCU-3. RAW 264.7 cells were transfected with siRNAs for 24 h and then stimulated with R848 for 24 h. Lipofectamine RNAiMAX Reagent (Invitrogen, Waltham, MA, USA) was used to transfect siRNA duplex into Raw 264.7 cells, according to the manufacturer's instructions.

### Transmission electron microscopy

Cells were fixed with 2.5% glutaraldehyde overnight at 4 °C and then washed three times in PBS. As for postfixation, 1% OsO_4_ solution was used to incubate samples for 1 h. After dehydration in an ethanol gradient (55, 75, 95% for 15 min each, 100% for 20 min), the samples were then embedded in Epon 812. Sections were then stained with 2% uranylacetate for 20 min and lead citrate for 5 min, and then examined with a TECNAI 10 transmission electron microscope (Phillips, Amsterdam, Netherlands).

### Immunofluoresence and HE staining

RAW 264.7 cells grown on coverslips were transfected with the pcDNA3.1- GFP- LC3 plasmids using the X-treme GENE HP DNA transfection reagent as described above and then the cells were unstimulated or stimulated with 0.1 *μ*g/ml R848 for 24 h subsequently. After fixation with 4% paraformaldehyde in PBS, the coverslips were mounted on slides and viewed on a fluorescence microscope (Nikon eclipse Ti, Tokyo, Japan). RAW 264.7 cells grown on coverslips were unstimulated or stimulated with 0.1 *μ*g/ml R848 for 24 h. After fixation and permeabilization, cells were incubated in blocking buffer (5% BSA in PBS, contain 0.1% Triton X-100) for 1 h. Cells were then incubated with mouse anti-cytochrome c antibody (Santa Cruz, Dallas, TX, USA) overnight at 4 °C. Subsequently, cells were washed and stained with Alexa Fluor 594 conjugated goat anti-mouse IgG secondary antibody (abcam) for 1.5 h. The nucleus were stained with DAPI in dark for 10 min and washed with PBS for three times. Finally, the coverslips were mounted on slides and examined under the FV10iconfocal microscope (OLYMPUS, Tokyo, Japan). For the histopathological analysis, murine renal tissues were fixed in 4% paraformaldehyde and embedded in paraffin. The sections (4 mm) were post stained with hematoxylin and eosin according to the according to the manufacturer's instructions (Beyotime). Histopathologic findings of the kidneys were graded in a blinded manner, on a scale of 0–3, as previously reported.^52^

### RNA extraction and quantitative real-time PCR

Total RNA was isolated using Trizol Reagent (Invitrogen) according to the manufacturer's instructions. Real-time PCR assay was then performed using SYBR green dye (Invitrogen) on StepOne sequence detection system (Applied Biosystems, Waltham, MA, USA). Relative abundance of genes was calculated by using 2^−ΔΔCT^ formula, with GAPDH as an internal control.

### Western blot

Proteins were exacted with an appropriate volume of lysis buffer. After electrophoresis on SDS-PAGE, proteins were transferred onto PVDF membranes (Millipore). Antibodies used here were anti-LC3I/II (Sigma), anti-P62 (Proteintech), anti-notch1 (Cell Signaling Technology), anti-Hes-1 (Santa Cruz), anti-GAPDH (Cell Signaling Technology), anti-Tublin (Cell Signaling Technology) and goat anti-rabbit IgG HRP (Cell Signaling Technology).

### Flow cytometry assay

For the surface marker staining, cells were stained according to the manufacturer's directions. The following antibodies were used: anti-F4/80 (eBioscience), anti-CD80 (eBioscience, Waltham, MA, USA), anti-CD86 (eBioscience), anti-MHCII (eBioscience). To detect LC3II in splenic macrophages, Flowcellect autophagy LC3 antibody-based assay kit (Merk Millipore) was used according to the manufacturer's directions. To assess TLR7, Notch1 expression in macrophages, spleen cells were then stained with anti-F4/80 and resuspended with fixation/permeabilization solution (eBioscience) then stained with anti-TLR7 (IMGENEX, CO, USA), anti-Noch1 (eBioscience), respectively. Similarly, fixed and permeabilized cells were stained with anti-Hes-1 (abcam) or anti-P62 (Merk Millipore), and then stained with Alexa Fluor 647 conjugated goat anti-rabbit IgG secondary antibody (abcam) and Alexa Fluor 488 conjugated goat anti-mouse IgG secondary antibody (abcam), respectively. Annexin V-FITC and PI Apoptosis kit (eBiosciences) was used to examin the mortality of macrophages *in vivo* and *in vitro*. All of the flow cytometry data were aquired with the BD FACS Calibur cytometer and analyzed by FlowJo software.

### Statistical analysis

All of the values presented on the graphs are given as means ±S.E.M. ANOVA and unpaired Student's *t*-tests were used to analyze the statistical significance, and *P*-values <0.05 were considered statistically significant.

## Abbreviations

SLE: systemic lupus erythematosus
TLR7: toll-like receptor 7
SNP: single-nucleotide polymorphism
ATG5: autophagy-related gene 5
BMDM: bone marrow-derived macrophages
IMQ: imiquimod
Hes-1: Hairy and enhancer of split 1
Hey-1: hairy/enhancer of split related with YRPW motif 1
Notch1IC: Notch1 intracellular domain
P62/SQSTM1: sequestosome 1
Cyt c: cytochrome c
3-MA: 3-methyladenine
DAPT: N -[N-(3,5-difluoroph enacetyl)-L-al anyl]-S-phenylglycine t-butyl ester
TEM: transmission electron microscopy
