# Supplementary material for: Notch-Hes-1 axis controls TLR7-mediated autophagic death of macrophage via induction of P62 in mice with lupus
Source: Cell Death Dis. 2016 Aug 18;7(8):e2341–. doi: 10.1038/cddis.2016.244 (PMC5108329; doi:10.1038/cddis.2016.244)

Figure S1. Marked splenomegaly and activation of lymphocytes in IMQ-treated mice.

(A) IMQ-treated mice at 10 weeks showed marked splenomegaly (B) Weight of spleens from IMQ-treated mice and C57BL/6 control mice. (C, D) Increased expression of CD69 in B cells (C) and T cells (D) from IMQ-treated mice. Representative histograms are on the right. (B-D) The data are shown as means ± SEMs and represent 2 independent experiments. *P values <0.05, **P<0.01 and ***P<0.001, determined by t tests.

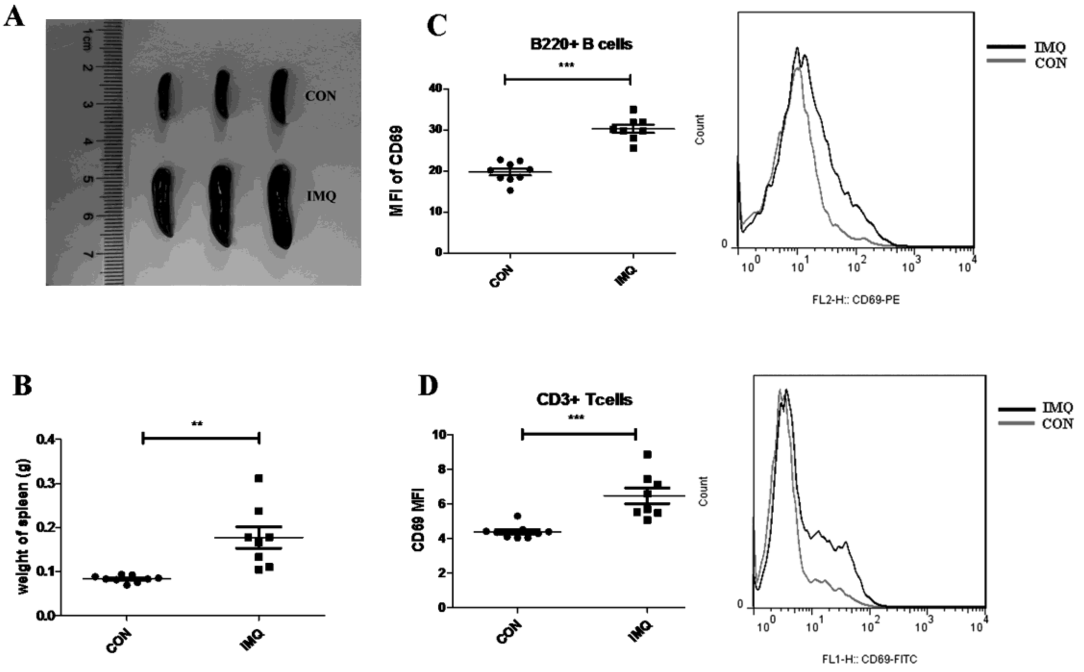

Figure S2. Severe glomerulonephritis and proteinuria in IMQ-treated mice.

(A) HE staining of renal tissues from C57BL/6 control mice (left) and IMQ-treated mice (B) Histopathologic scores in C57BL/6 control mice and mice treated with IMQ. (C) Proteinuria in C57BL/6 control mice and IMQ-treated mice. (B, C) The data are

shown as means ± SEMs and represent 2 independent experiments. *P values <0.05, **P<0.01and ***P<0.001, determined by t tests.

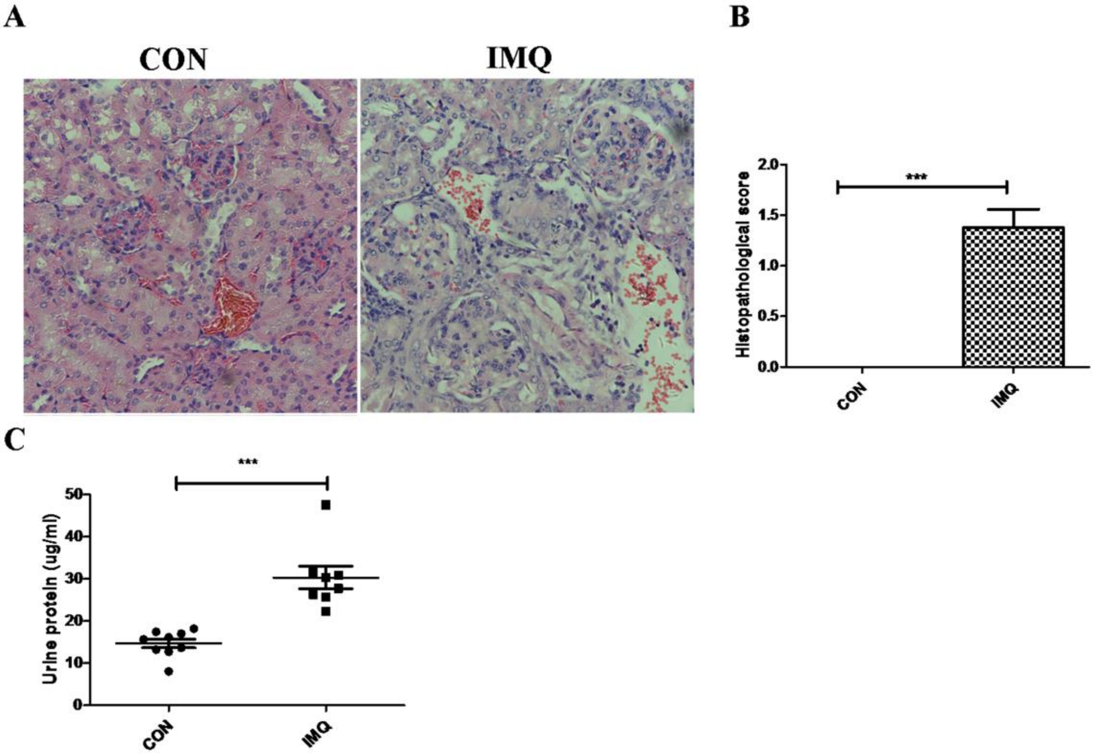

Supplement: Supplementary Figures [file cddis2016244x1.pdf]
